# Pleura-ABCDE - a structured expert-based protocol for neonatal lung ultrasound documentation and interpretation

**DOI:** 10.1186/s13089-025-00442-4

**Published:** 2025-10-06

**Authors:** Jan Sandig, Erik Küng, Lukas Aichhorn, Bernhard Schwaberger, Mathias Klemme, Natascha Wagner, Christoph Bührer, Fabian Sternal

**Affiliations:** 1 Division of Pediatric Radiology, Charité - Universitätsmedizin Berlin, Berlin, Germany; 2 Department of Neonatology, Charité - Universitätsmedizin Berlin, Berlin, Germany; 3 Division of Neonatology, Pediatric Intensive Care & Neuropediatrics, Department of Pediatrics and Adolescent Medicine, Comprehensive Center for Pediatrics, Medical University of Vienna, Vienna, Austria; 4 Division of Neonatology, Department of Pediatrics and Adolescent Medicine, Medical University of Graz, Graz, Austria; 5 Division of Neonatology, Dr. v. Hauner Children’s Hospital and Perinatal Center Munich - Grosshadern, Ludwig-Maximilians-University Munich, Munich, Germany; 6 Institut für Notfallmedizin und Medizinmanagement (INM) Ludwig-Maximilians- University Munich, Munich, Germany; 7 Medical Faculty, Department of Neonatology, Johannes Kepler University, Kepler University Hospital, Linz, Austria

**Keywords:** Point-of-care ultrasound, Lung ultrasound, Ultrasound, Pleura-ABCDE, Neonate

## Abstract

**Background:**

Neonatal lung ultrasound is a rapidly emerging imaging modality with increasing impact, but lacks standardized protocols and curricula, resulting in inconsistent dissemination and quality assurance.

**Results:**

We present a structured protocol for documentation and interpretation of lung ultrasound. For each lung region, the acronym Pleura-ABCDE is used to analyze the pleura (sliding, morphology), A-Lines, B-Lines, Consolidation, Dynamics (lung point, double lung point) and Effusion. The structured documentation and interpretation could provide clues to differentiate respiratory diseases in newborns.

**Conclusion:**

The Pleura-ABCDE protocol provides structured documentation and interpretation support for lung ultrasound in neonates. In contrast to flowchart-based protocols, a pattern-based approach and link to clinical presentation allows an integrative perspective on the use of neonatal lung ultrasound. Therefore, with this expert-based proposal, we aim to improve documentation and thereby support diagnostic quality and reproducibility, as recommended by international ultrasound societies.

**Supplementary Information:**

The online version contains supplementary material available at 10.1186/s13089-025-00442-4.

## Background

Lung ultrasound is a rapidly growing methodology that enables the clinician to gain bedside real-time information and compare the results to clinical findings. The neonatal lung ultrasound examination is a unique technique with specific features, indications and diseases. The small size of the diagnostic surface results in a fast examination time and the utilization of high-resolution ultrasound probes. Therefore, lung ultrasound has the potential to speed up diagnostics, enable follow-up examinations and reduce the risk of radiation exposure for our vulnerable neonatal patients [1].

Although a promising steep learning curve in beginners [2, 3] and a high interobserver agreement [4], the disadvantages (as for all ultrasound examinations) of lung ultrasound are the dependency on the experience and level of training of the investigators as well as the physical constraints of the ultrasound technique. In light of these challenges, international ultrasound societies have advocated for the necessity of standardization, training programs, and Point-of-Care Ultrasound Stewardship initiatives [5]. Recent progress in lung ultrasound publications try to set up a standardization for examination protocols, result interpretation and terminology [5–9]. Furthermore, significant advances have been made in the differentiation of unique ultrasound patterns, which hold the potential to guide clinicians in differentiating respiratory pathologies [8, 10] and offer a decision-making tool, particularly in the emergency setting [11, 12].

Despite the growing body of evidence, there remains a paucity of standardized report forms and educational curricula specifically designed for the ultrasound examination of the neonatal lung [13].

We introduce a pragmatic, hands-on approach to neonatal lung ultrasound, which includes a standardized documentation protocol (the Pleura-ABCDE protocol) to establish a benchmark for quality assurance and improve the comparability and communication of lung ultrasound examinations. This approach could also guide clinicians in the interpretation of ultrasound findings, facilitating the differentiation of respiratory diseases in newborns.

This protocol reflects the shared expert opinion of a group of 8 clinicians, representing five hospitals in two countries (4 clinicians from Austria, 4 clinicians from Germany) each with more than five years of experience in neonatal lung ultrasound. While no formal consensus methodology (e.g., Delphi or anonymized voting) was applied, the recommendations were developed through collaborative online discussions. These discussions aimed to integrate available evidence with practical insights from clinical experience to formulate a pragmatic and consistent approach to neonatal lung ultrasound in addition to a targeted literature search by five author group members [14]. The consensus, reached through online conferences, synthesized evidence and expert insights to ensure a standardized and reliable approach. Artificial intelligence (DeepL Write, ChatGPT) was used for text editing and proofreading. The final content has been created following a critical revision and approval by all authors, to ensure the scientific integrity and prevent ethical ambiguity.

## Results

### Pleura-ABCDE protocol

**Fig. 1:**
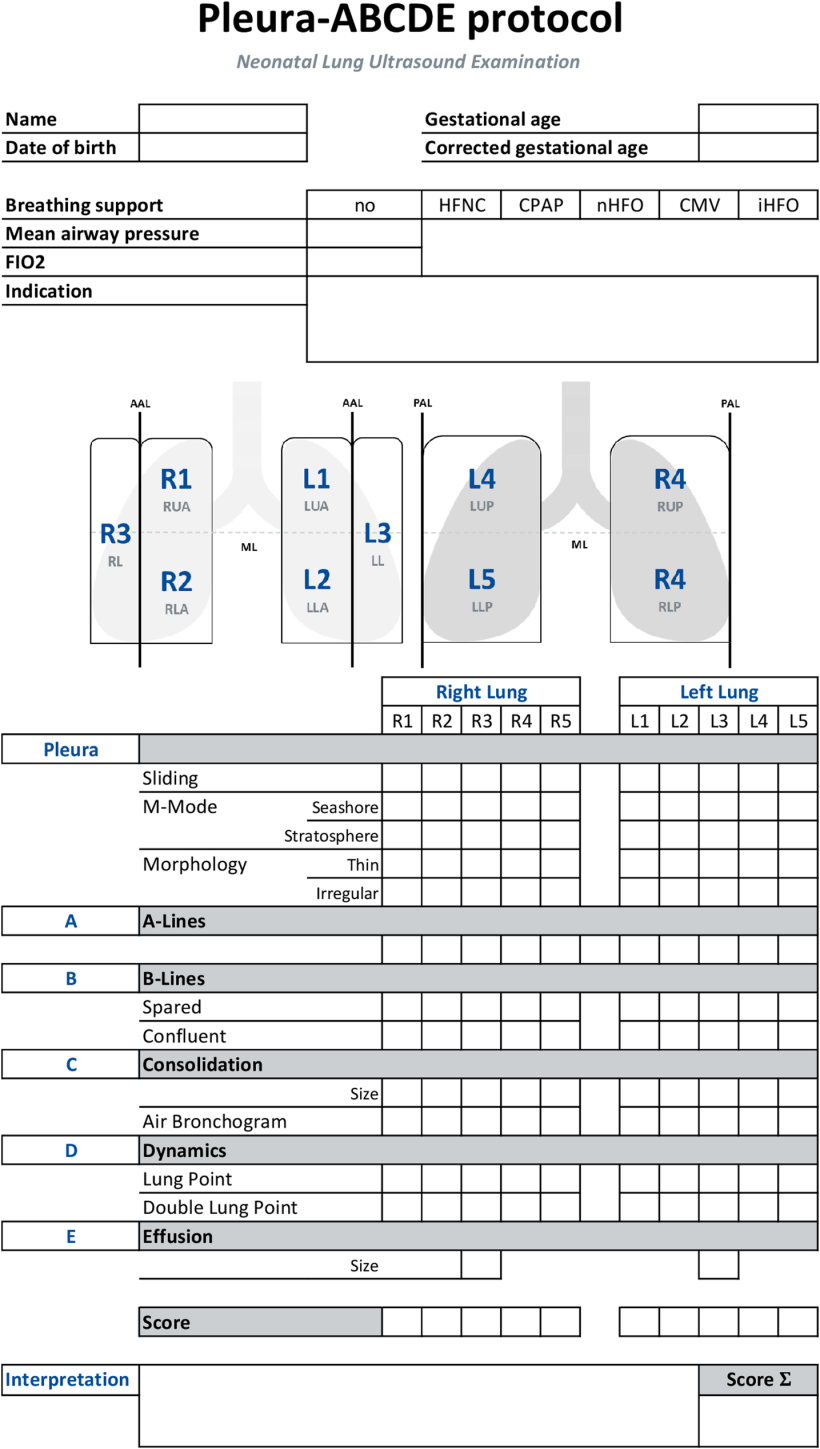
Pleura-ABCDE protocol, FIO2– fraction of inspired oxygen, HFNC– High flow nasal cannula, CPAP– Continuous positive airway pressure, nHFO– non-invasive high-frequency oscillation, CV– Conventional mechanical ventilation, iHFO– invasive high-frequency oscillation, LUA– left upper anterior region, LLA– left lower anterior region, LL– left lateral region, LUP– left upper posterior region, LLP– left lower posterior region, RUA– right upper anterior region, RLA– right lower anterior region, RL– right lateral region, RUP– right upper posterior region, RLP– right lower posterior region

The Pleura-ABCDE protocol is presented in Fig. 1. Prior to the examination, patient data such as name, date of birth, gestational age and corrected gestational age are documented. Furthermore, the indication for the examination, the breathing support and the fraction of inspiratory oxygen are noted.

In line with international guidelines, we recommend a 10-zones approach to separate the lungs into five partes for each lung for the documentation and assessment of lung artefacts [9, 15].

In a complete lung examination, all lung areas are thoroughly scanned in the sagittal plane during a minimum of three breathing cycles, moreover a transversal / intercostal scan could be added and the transdiaphragmatic view could be used for the detection of pleural effusions. As a standard form of minimum documentation, it is recommended that a video clip (duration of at least three seconds) of every lung area is saved in the sagittal plane. During the examination, every lung area should be analyzed, and the artefacts are documented using the following Pleura-ABCDE protocol.

In critically ill or unstable neonates, Point-of-Care ultrasound should be used as a targeted Point-of-Care toolkit. Rather than completing a full protocol in all cases, the examination should prioritize relevant findings while minimizing stress for the patient.

To complete the Pleura-ABCDE protocol, a brief overview of the key ultrasound findings included is provided.

### Pleura

**Fig. 2:**
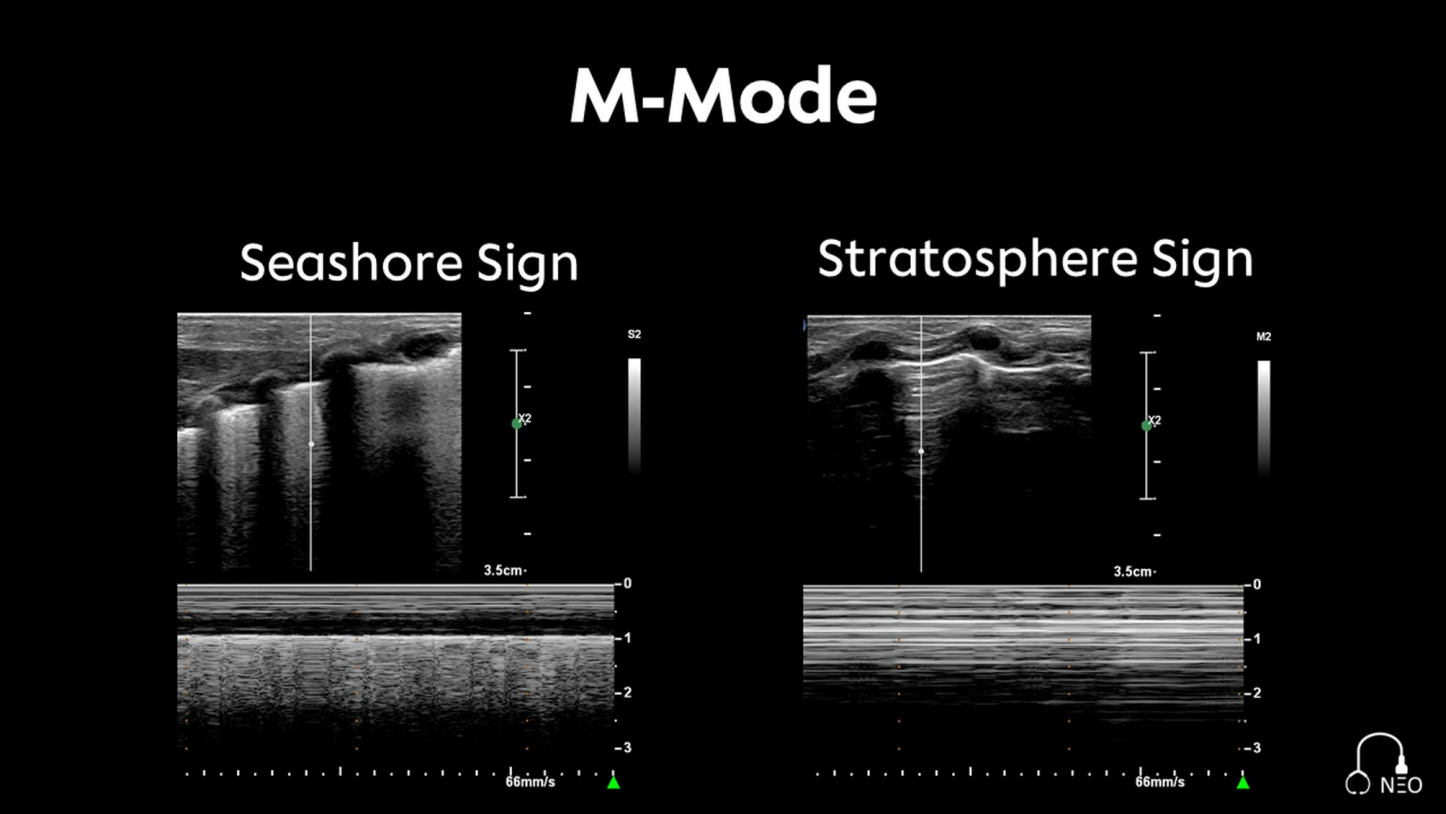
M-Mode Seashore sign and Stratosphere sign

**Fig. 3:**
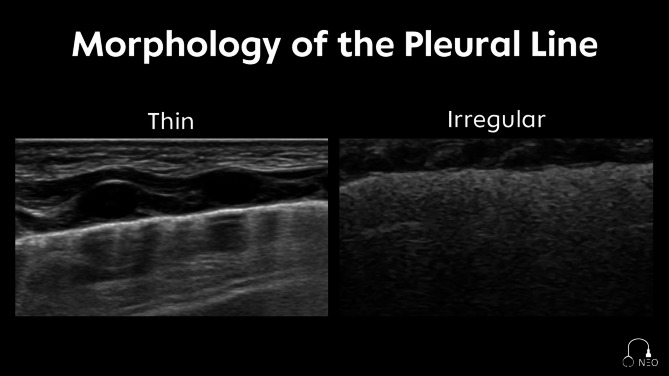
Morphology of the pleural line

The pleura appears as an echogenic horizontal line beneath the ribs and intercostal muscles. Two aspects of the pleural line are significant for diagnostic assessment:

1. Lung sliding: Visualization of a respiratory-synchronous movement of the visceral and parietal pleura. Lung sliding can be made visible in M-Mode by documenting the so-called Seashore sign. No lung sliding visualizes the Stratosphere sign:
  The *Seashore sign* appears as horizontal echogenic lines (waves) representing muscle and fat tissue above a homogeneous artifact structure (beach) due to the moment of the lung and the creation of artifacts (Fig. 2). This finding indicates regular ventilation of the lungs [16].
  The *Stratosphere sign* indicates the disappearance of motion artefacts and the creation of a barcode-like appearance due to the emergence of constant horizontal A-Lines (Fig. 2). The presence of the Stratosphere sign may suggest the possibility of a pneumothorax [16].
2. Pleural line: The pleural line can appear regular (thin and well-defined) or irregular (often mentioned as rough or thickened), with a blurred and partially interrupted appearance (Fig. 3). The thickening of the pleural line changes within the respiratory cycle and is increased in respiratory distress syndrome and neonatal acute respiratory distress syndrome compared to patients without lung pathologies [17].

### A-Lines

**Fig. 4:**
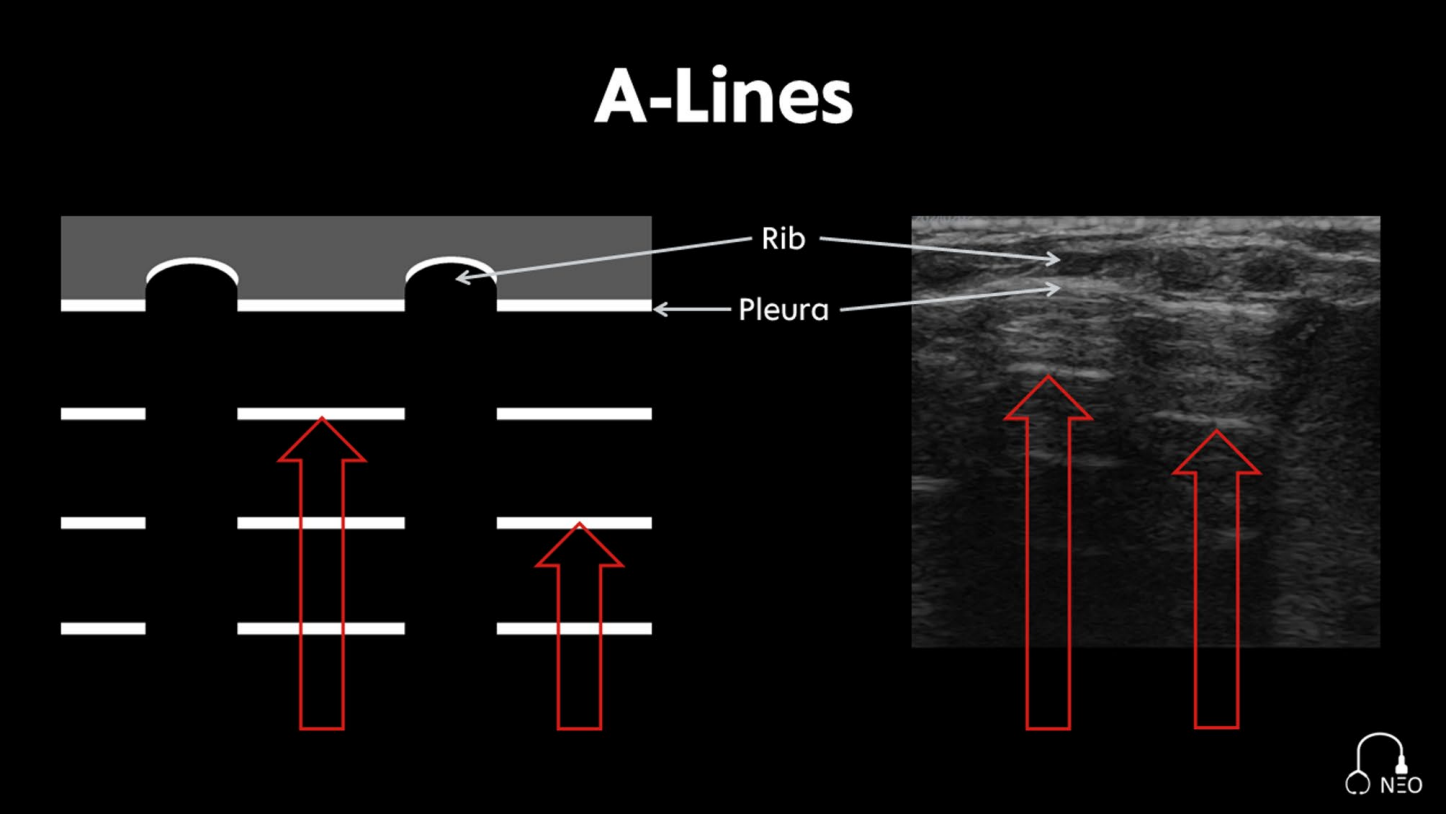
A-Lines

Echogenic horizontal reverberation artifacts, which extend repeatedly into the depth of the image at equal distances between the transducer and the pleura (Fig. 4).

### B-Lines

**Fig. 5:**
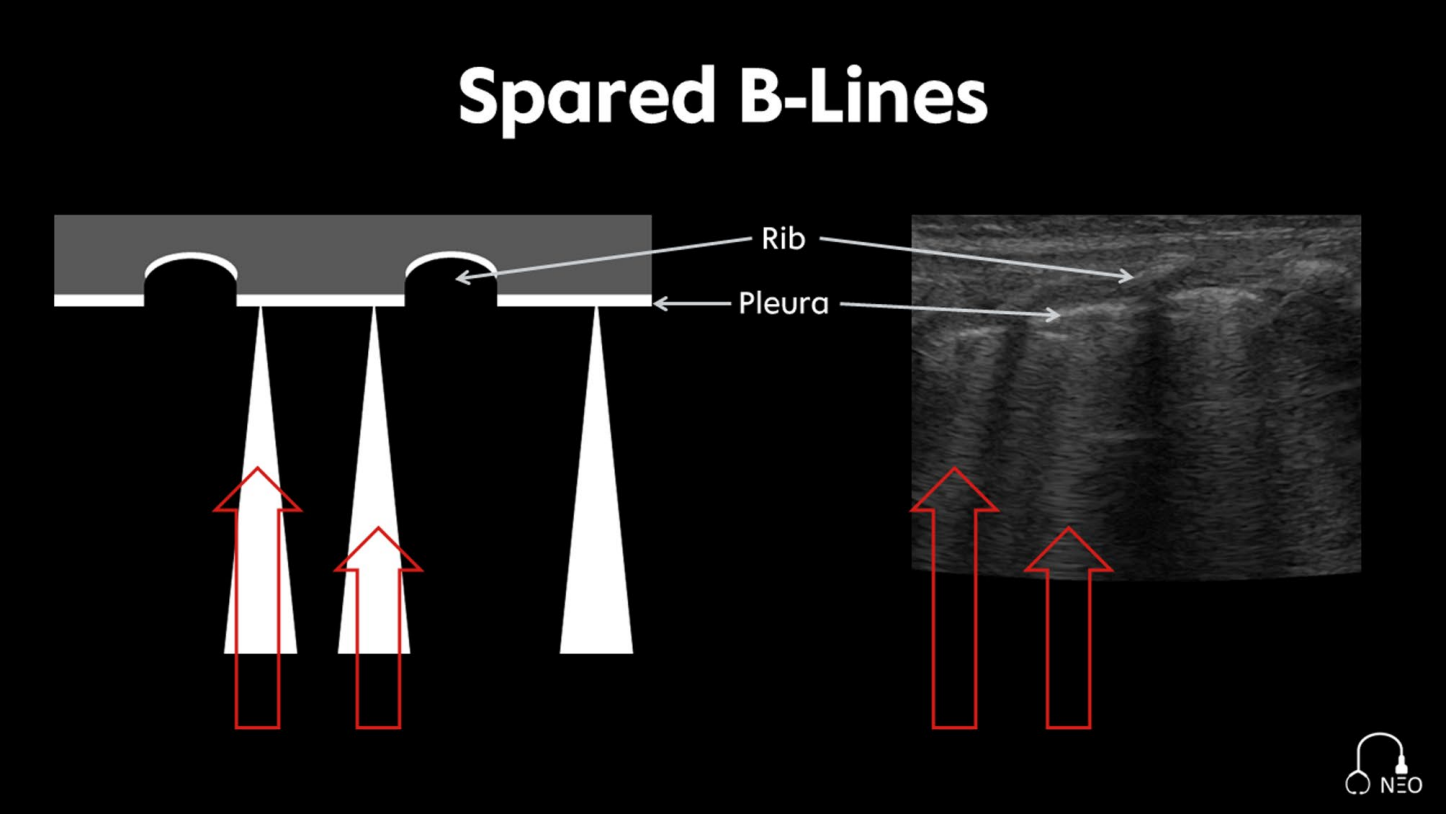
Spared B-Lines

**Fig. 6:**
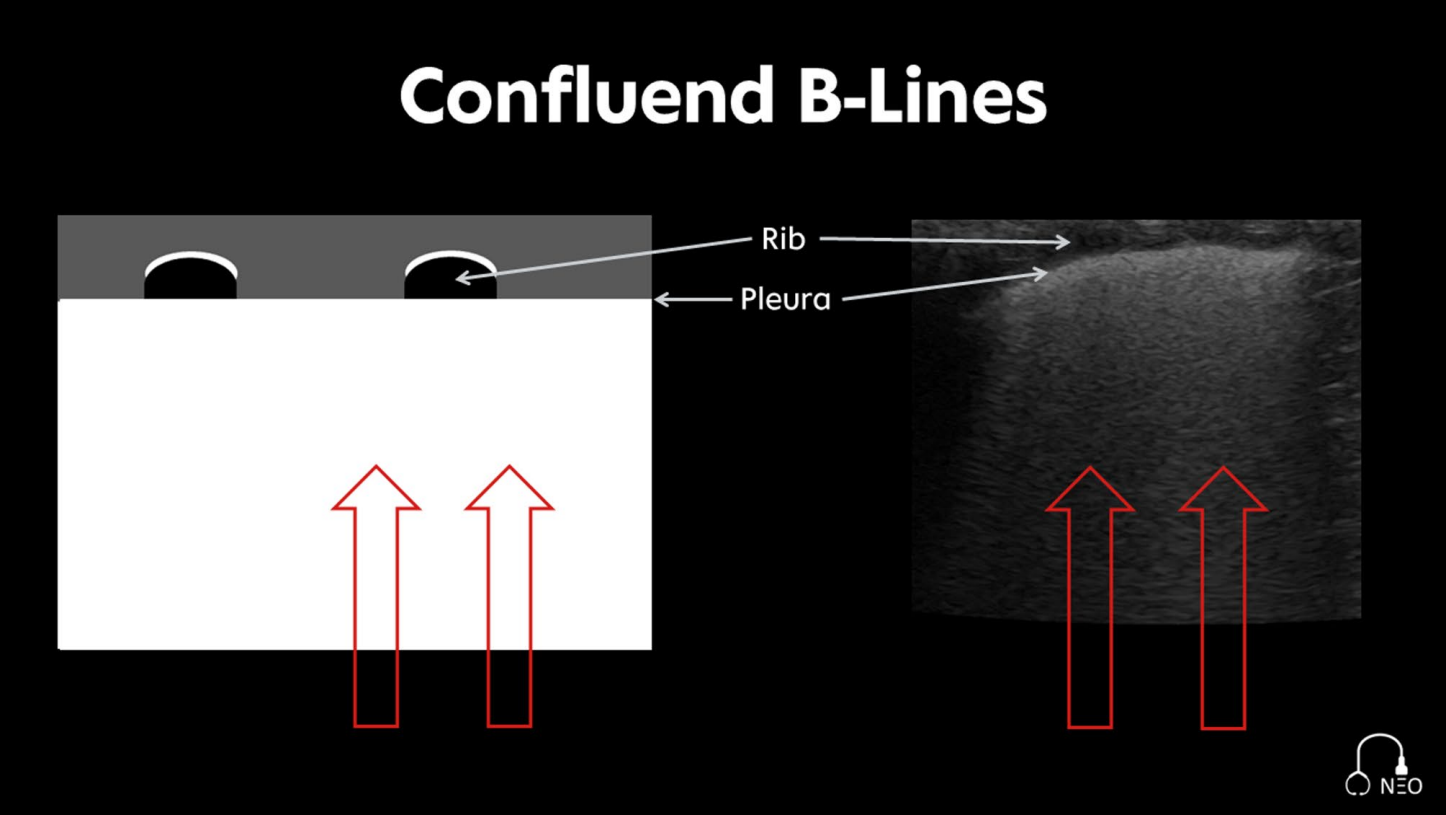
Confluent B-Lines

B-Lines are vertical echogenic “laser-like” ring-down artefacts that originate from the visceral pleura and exhibit a respiratory-synchronous horizontal movement [18]. B-Lines partially erase A-Lines. Literature distinguishes several different types of B-Lines. For reasons of simplicity, we differentiate between two forms:

1. *Spared B-Lines*: Isolated B-Lines in the intercostal space [19] (Fig. 5).
2. *Confluent B-Lines*: Filling the entire intercostal space; “white lung” [19] (Fig. 6).

### Consolidation

**Fig. 7:**
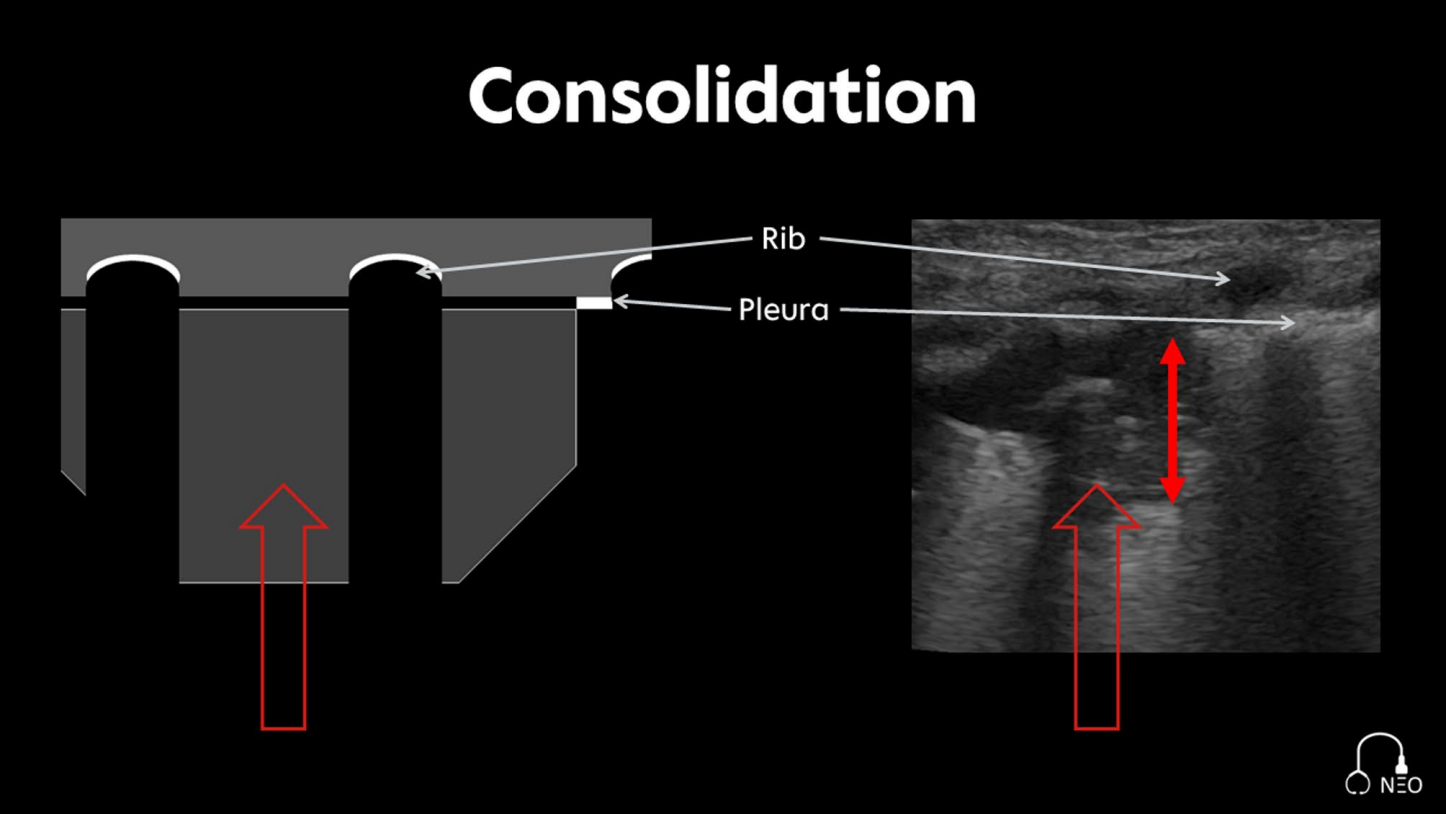
Consolidation, the double-headed red arrow indicates the measurement of the consolidation size (consolidation index)

Consolidations are characterized as hypoechoic tissue-like structures, originating from the pleura (Fig. 7) [20]. Their extent is typically quantified by measuring the vertical distance from the pleural surface to the deepest edge of the lesion (consolidation index) as described in the UNION International multicenter study on neonatal respiratory failure [21]. To distinguish true consolidations from subpleural consolidations, a threshold depth of ≥ 0.5 cm/kg or > 1 cm is applied. Lesions below this threshold are generally considered pleural irregularities or part of an interstitial pattern and are not classified as consolidations.

**Fig. 8:**
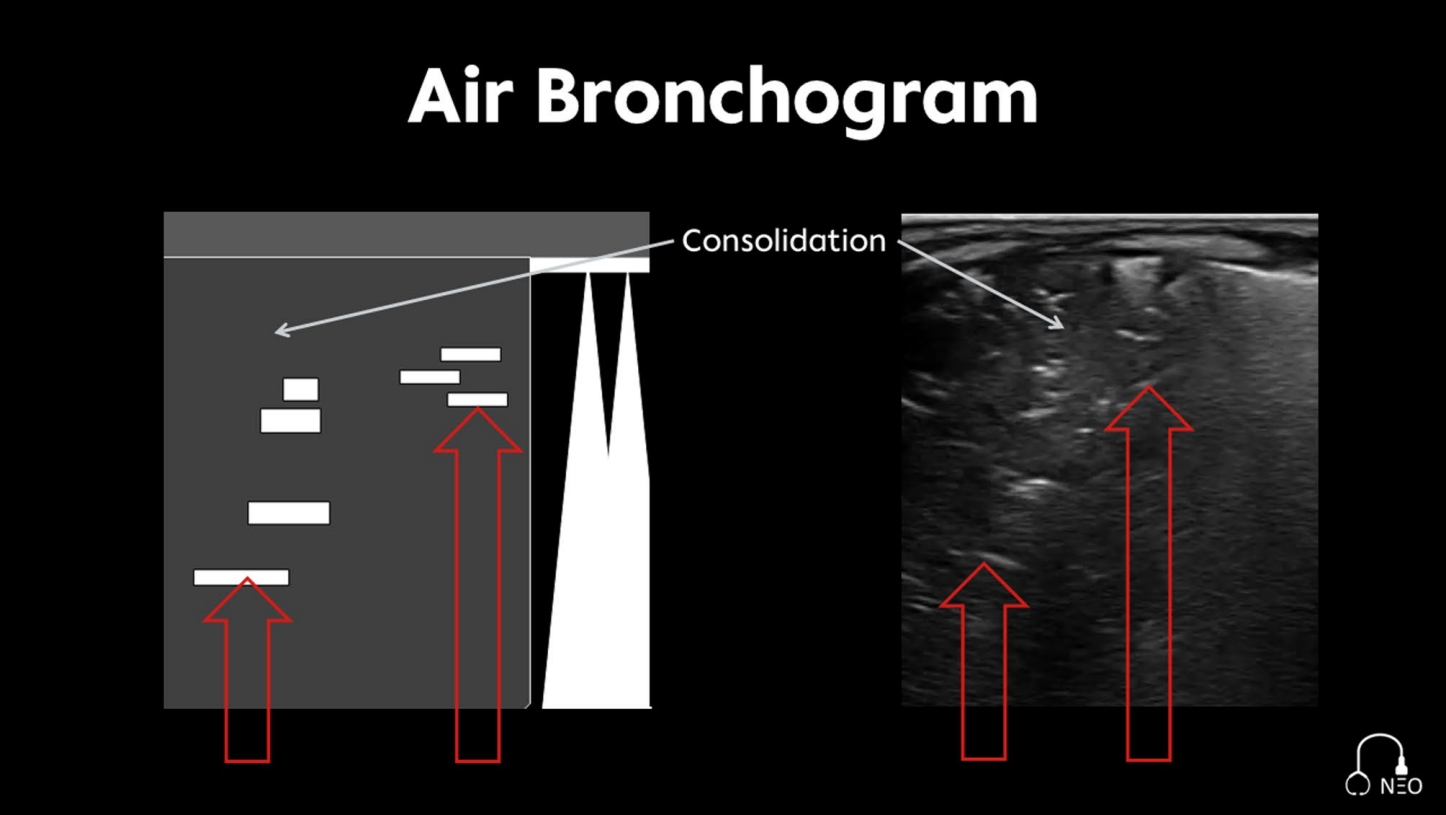
Air bronchogram

Within the consolidation echogenic artefacts might be visualized as air bronchogram (Fig. 8) [22]. It is caused by air trapping in a structure devoid of air.

### Dynamics

#### Lung point

**Fig. 9:**
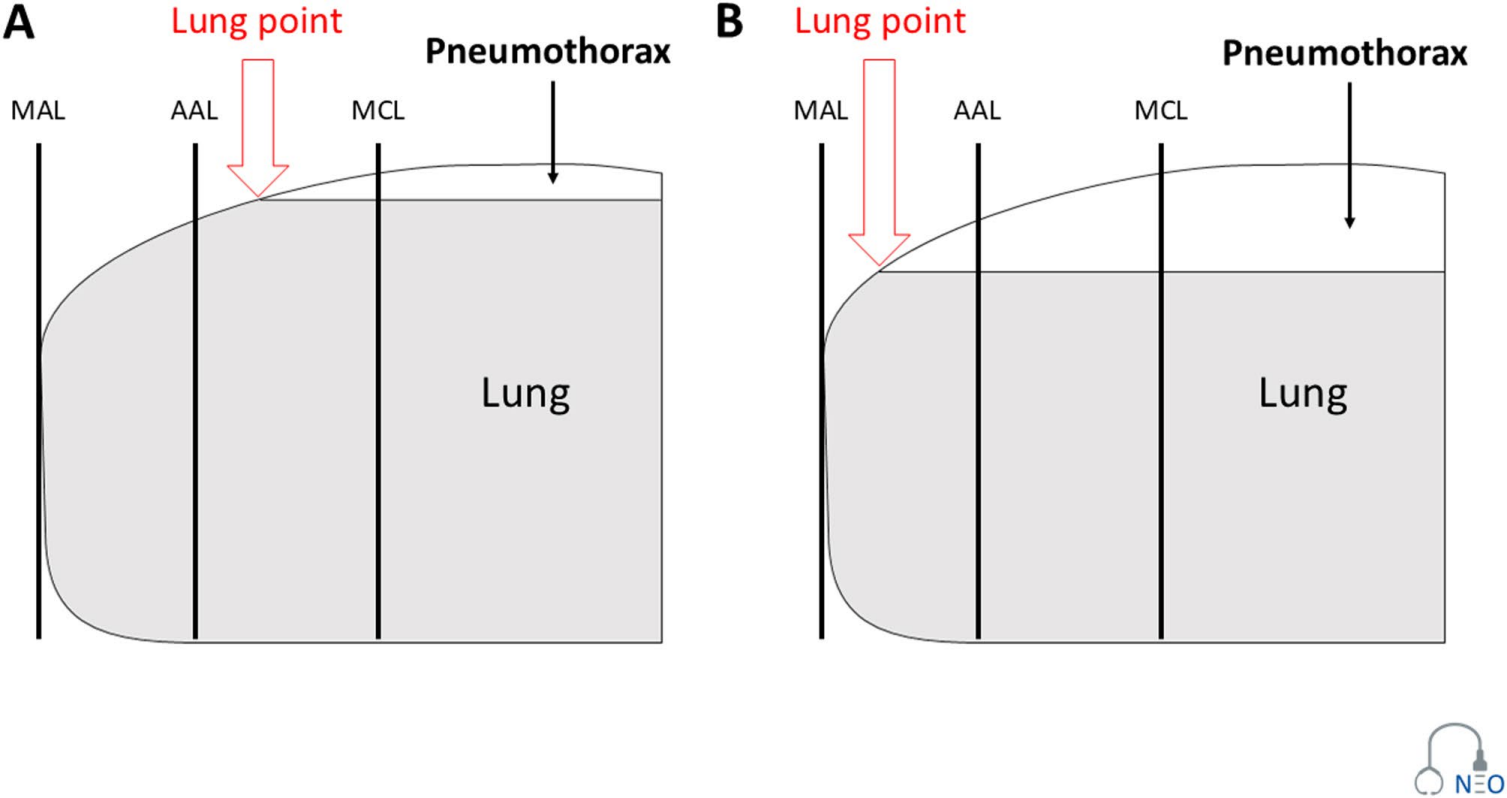
Lung point, MAL - Mid axillary line, AAL - Anterior axillary line, MCL - Mid clavicular line

The Lung point is the pathognomonic sign for the presence of a pneumothorax [23]. The term is used to describe the point at which the visceral and parietal pleura are in direct contact on one side (pleural sliding is visible, B-Lines are present) and on the other side, the two pleural layers are separated by air/pneumothorax (pleural sliding is not visible, B-Lines are absent, Fig. 9A). In the supine position of the neonate, air is usually accumulated at the anterior region of the thorax. The documentation of the anatomical region (mid clavicular line, anterior axillary line, mid axillary line, posterior axillary line) and the dynamic change could support the estimation of the size of a pneumothorax [24]. In case of an increasing pneumothorax, the lung point is shifted to the lateral thoracic wall. (Fig. 9B).

#### Double lung point

**Fig. 10:**
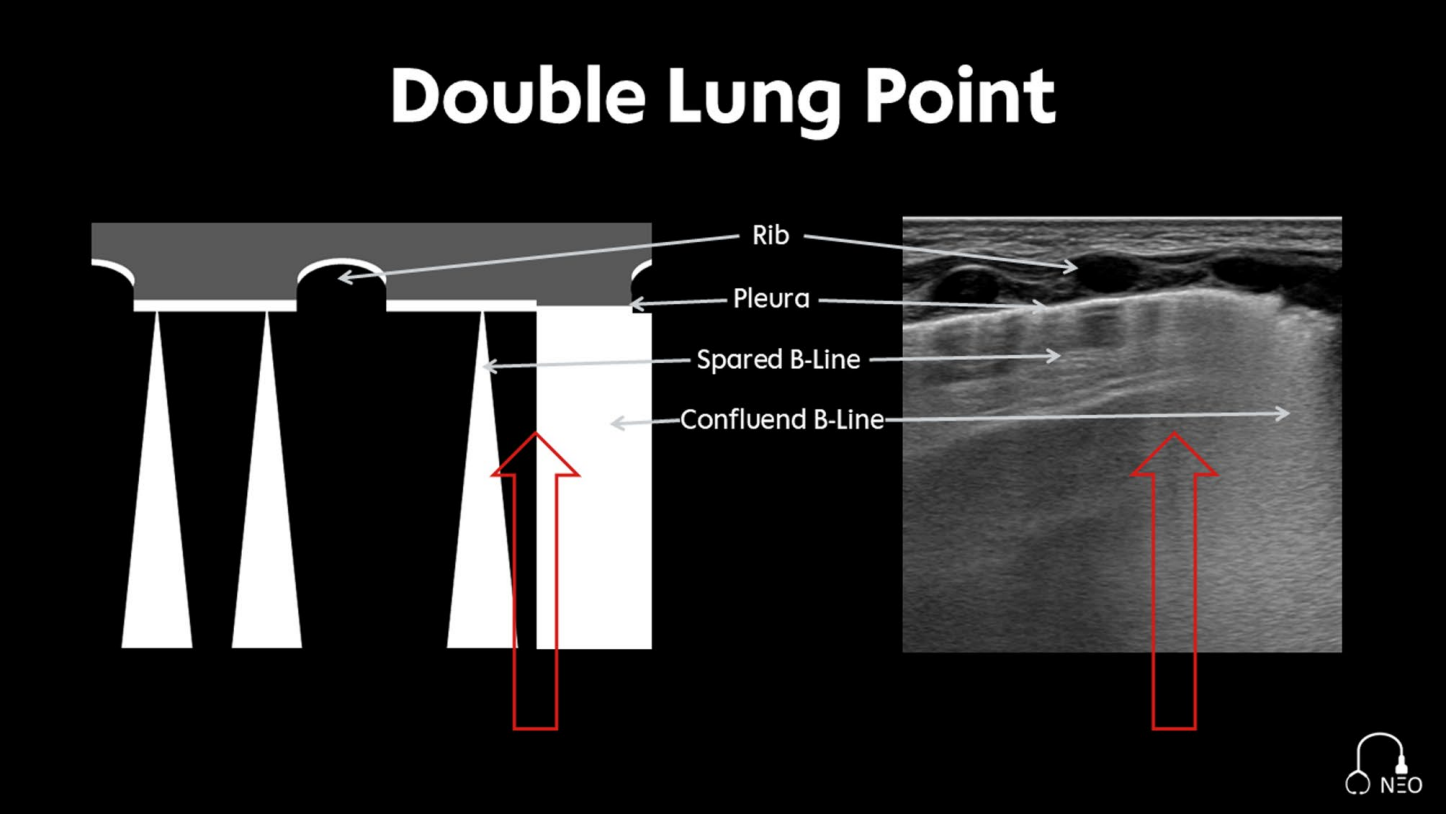
Double lung point

The Double lung point (Fig. 10) is used to denote a discernible discrepancy in the sonographic image, manifesting as confluent B-Lines in the inferior lung regions and spared B-Lines or A-Lines in the superior lung regions [25]. This finding may be indicative of an inhomogeneous water clearing process, which is more pronounced in the superior or ventral lung regions. This phenomenon has been observed in transient tachypnoea of the newborn [25].

### Effusion

Pleural effusion refers to an accumulation of fluid in the pleural space. Following the force of gravity, the largest amount of fluid in newborns positioned in the supine position can therefore be detected in the costodiaphragmatic recess, approximately at the level of the posterior axillary line in the sagittal plane.

**Fig. 11:**
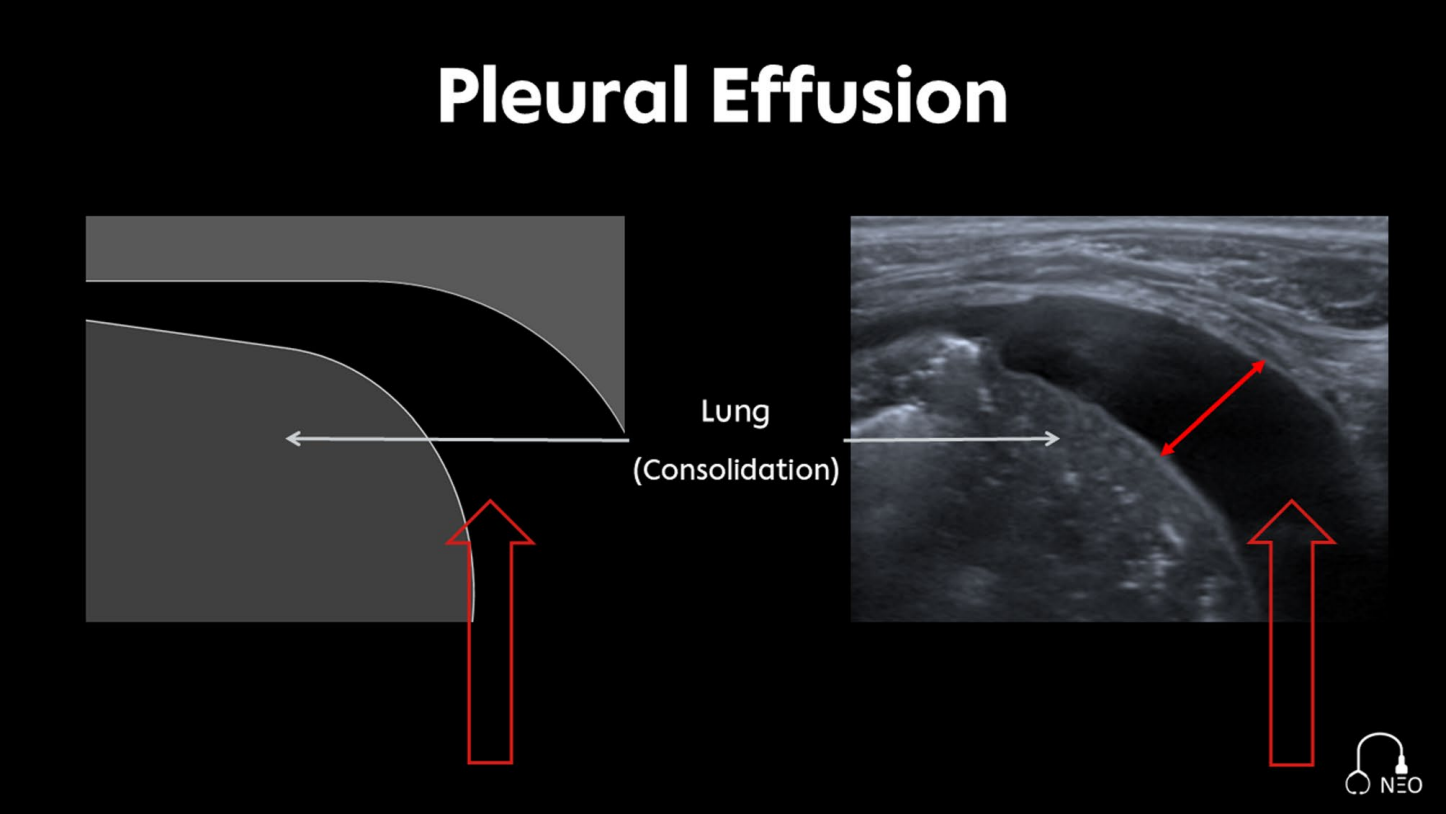
Pleural effusion, the double-headed red arrow indicates the measurement of the pleural effusion in the transversal plane

The measurement of pleural effusion should be performed at the site of a possible puncture and documented accordingly to allow for repeated measurements and comparability. We suggest measuring the effusion in the 4th intercostal space in the transversal (intercostal) plane measuring the maximum size between the lung surface and thoracic wall (Fig. 11).

### Score

There are numerous pulmonary scoring systems utilized for the guidance of clinical decision-making, e.g. surfactant therapy [10, 26, 27], the assessment of respirator weaning [28] or the evaluation of the severity of a respiratory illness [29]. Each scoring system is based on the sum of individual lung area scores, which could be added, based on the documentation of the Pleura-ABCDE protocol.

## Interpretation

Lung ultrasound is a useful diagnostic technology that can be used as a bedside tool by the treating physician. To improve the clinical impact of the Pleura-ABCDE protocol an interpretation guideline is demonstrated (Table 1). The authors wish to highlight that Table 1 offers a practical overview of typical lung ultrasound findings observed in defined thoracic regions and may support clinical reasoning in the context of neonatal respiratory distress. Nonetheless, it is essential to note that this table is neither comprehensive nor definitive. Considerable overlap between different pathologies is common, and distinguishing features are often indistinct or lacking. For this reason, isolated interpretation of sonographic findings should be avoided. A reliable diagnosis requires integration of additional modalities, including laboratory results, clinical features (e.g., timing of symptom onset, evidence of pulmonary hemorrhage), and relevant perinatal history such as gestational age or prolonged rupture of membranes.

**Table 1:**
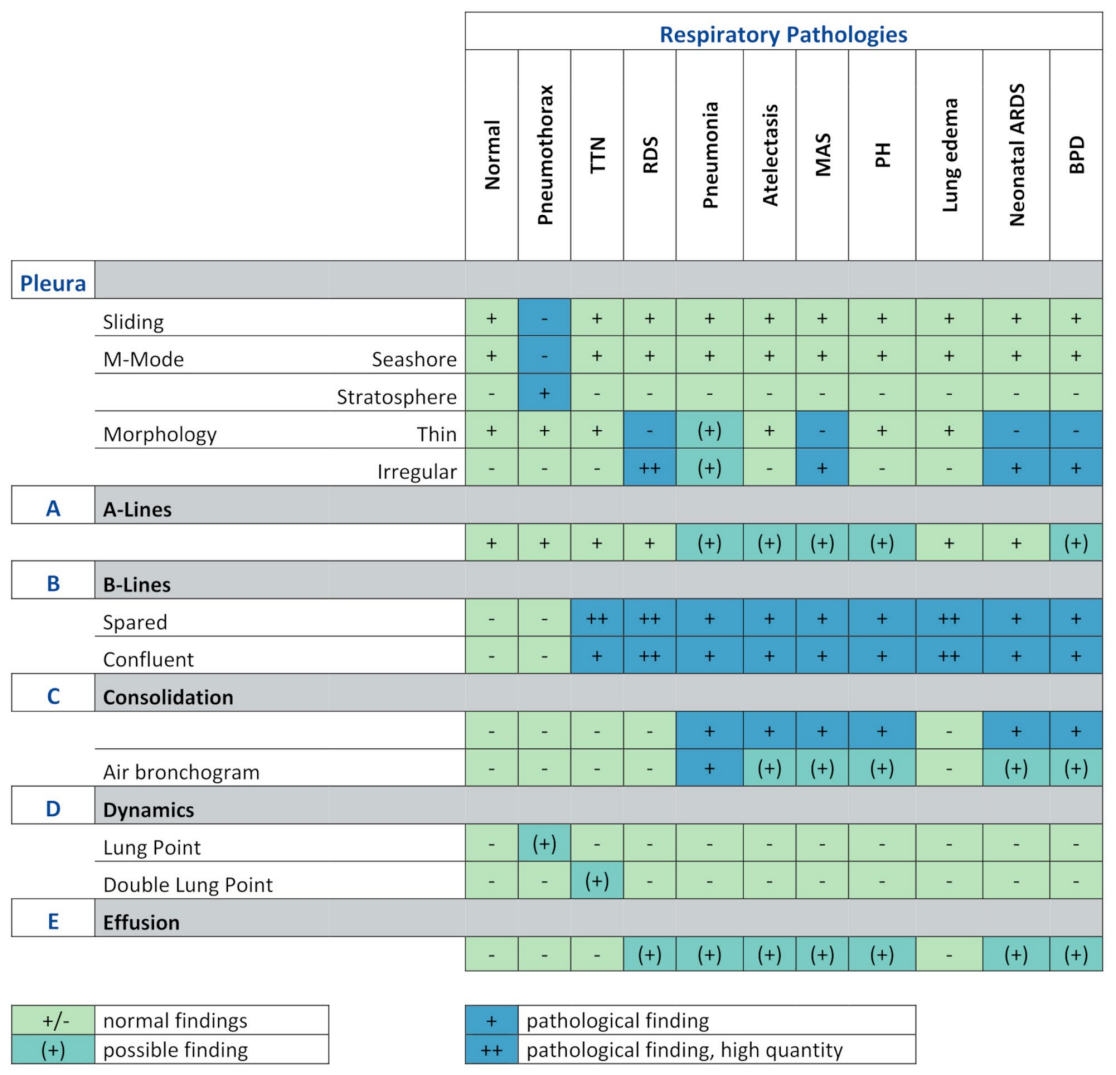
Pleura-ABCDE interpretation of respiratory pathologies of the newborn. *TTN– Transient tachypnoea of the newborn*,* RDS– Respiratory distress syndrome*,* MAS– Meconium aspiration syndrome*,* PH– Pulmonary hemorrhage*,* ARDS– Acute respiratory distress syndrome*,* BPD– Bronchopulmonary dysplasia*

For a detailed description of the specific ultrasound findings associated with respiratory diseases, we refer to previous publications and international expert consensus statements [30–32].

## Conclusion

The Pleura-ABCDE protocol represents an expert-based structured approach that proposes a standardized reporting and interpretation framework for clinicians. It is intended to support examination quality and reproducibility, aligning with goals expressed by international ultrasound societies [5, 33].

To address the absence of standardization guidelines, international groups dedicated to the field of ultrasound have proposed a series of standards for neonatal lung ultrasound examinations. The proposal involves the utilization of a documentation system based on an ABCDE framework [8]. The authors strongly support this approach, particularly its application as an extended lung ultrasound protocol. It is vital to highlight its user-friendliness and its practical usage. Consequently, an even more pragmatic approach has been adopted.

Through this structured guideline, all lung regions are systematically examined and classified to ensure a high level of reproducibility and quality assurance. The protocol is intended for the use as a universal tool of all neonatal patients without a difference in gestational age. This method contrasts with flow-chart-based approaches [12], where the clinician follows a pathway to rule-in or rule-out different diagnoses and could be used for all clinical scenarios (e.g. Emergencies or Follow-ups).

Lung ultrasound represents a significant advancement in modern neonatology, with the highest impact being achieved by integrating it with bedside examination tools, as opposed to traditional clinical examinations, laboratory diagnostics and bedside monitoring [34]. Ensuring the quality of ultrasound examinations necessitates that treating clinicians possess a comprehensive understanding of the indications and limitations of the examination, in addition to ensure a sufficient level of training. Based on our shared clinical and educational experience, we suggest that the Pleura-ABCDE protocol may serve as a useful component in training curricula, providing a structured approach to neonatal lung ultrasound.

Despite its primary clinical aim of supporting clinicians, the Pleura-ABCDE protocol could also be adapted and used as a standardized reporting tool in future research projects. To achieve this, the implementation of a multicenter prospective validation study could be implemented to assess reproducibility and diagnostic impact.

In conclusion, the Pleura-ABCDE protocol could enhance the systematic evaluation of lung ultrasound, facilitating rapid diagnosis and management of various pulmonary conditions. We believe this structured method may support clinical decision-making and promote the integration of ultrasound into routine neonatal care.

## Supplementary Information

Below is the link to the electronic supplementary material.

Supplementary Material 1

Supplementary Material 2

## Data Availability

The Pleura-ABCDE protocol and interpretation guideline is available in the supplement area. No data analyzation was used to create the manuscript.

## References

[1] Gordon PV, Swanson JR (2014) A simple step to reduce radiation exposure in the NICU. J Perinatol 34:331–332 24776600 10.1038/jp.2013.147

[2] Corsini I, Parri N, Gozzini E, Coviello C, Leonardi V, Poggi C et al (2019) Lung ultrasound for the differential diagnosis of respiratory distress in neonates. Neonatology 115:77–84 30304736 10.1159/000493001

[3] Mazmanyan P, Kerobyan V, Shankar-Aguilera S, Yousef N, De Luca D (2020) Introduction of point-of-care neonatal lung ultrasound in a developing country. Eur J Pediatr 179:1131–1137 32060800 10.1007/s00431-020-03603-w

[4] Goff CG-L, Vivalda L, Foligno S, Loi B, Yousef N, Luca DD (2020) Effect of different probes and expertise on the interpretation reliability of Point-of-Care lung ultrasound. Chest 157:924–931 31785252 10.1016/j.chest.2019.11.013

[5] Osterwalder J, Tabakovic S, Jenssen C, Dietrich CF, Connolly J, Polyzogopoulou E et al (2023) Emergency Point-of-Care ultrasound Stewardship– A joint position paper by EuSEM and EFSUMB and endorsed by IFEM and WFUMB. Ultraschall Med 44:379–388 36996862 10.1055/a-2041-3302

[6] Aichhorn L, Küng E, Schwaberger B (2023) Neonatologist performed lung ultrasound: NPLUS—proposal for a consistent ultrasound terminology. Front Pediatr 10:1007672 10.3389/fped.2022.1007672 PMC9971559 36866083

[7] Chidini G, Raimondi F (2024) Lung ultrasound for the sick child: less harm and more information than a radiograph. Eur J Pediatr 183:1079–1089 38127086 10.1007/s00431-023-05377-3

[8] Singh Y, Dauengauer-Kirliene S, Yousef N (2024) Setting the standards: neonatal lung ultrasound in clinical practice. Diagnostics (Basel) 14:1413 39001302 10.3390/diagnostics14131413 PMC11241677

[9] Mongodi S, Cortegiani A, Alonso-Ojembarrena A, Biasucci DG, Bos LDJ, Bouhemad B et al (2025) ESICM-ESPNIC international expert consensus on quantitative lung ultrasound in intensive care. Intensive Care Med. 51(6):1022-1049 10.1007/s00134-025-07932-y 40353867

[10] Raimondi F, Yousef N, Migliaro F, Capasso L, De Luca D (2021) Point-of-care lung ultrasound in neonatology: classification into descriptive and functional applications. Pediatr Res 90:524–531 30127522 10.1038/s41390-018-0114-9 PMC7094915

[11] Yousef N, Singh Y, De Luca D (2022) Playing it SAFE in the NICU SAFE-R: a targeted diagnostic ultrasound protocol for the suddenly decompensating infant in the NICU. Eur J Pediatr 181:393–398 34223967 10.1007/s00431-021-04186-w PMC8256195

[12] Kurepa D, Zaghloul N, Watkins L, Liu J (2018) Neonatal lung ultrasound exam guidelines. J Perinatol 38:11–22 29144490 10.1038/jp.2017.140

[13] Alonso-Ojembarrena A, Ehrhardt H, Cetinkaya M, Lavizzari A, Szczapa T, Sartorius V et al (2024) Use of neonatal lung ultrasound in European neonatal units: a survey by the European society of paediatric research. Archives Disease Child - Fetal Neonatal Ed 109:660–664 10.1136/archdischild-2024-327068 38604653

[14] Küng, E., Schwaberger, B., Gruber, V., Pramhofer, N., Klemme, M., Schneider, M., Habrina, L., Werther, T., & Aichhorn, L. (2024). Proposed Standard for Neonatologist Performed Lung Ultrasound (v5.8). Zenodo.; Available from: https://zenodo.org/records/13908117

[15] Loi B, Vigo G, Baraldi E, Raimondi F, Carnielli VP, Mosca F et al (2021) Lung ultrasound to monitor extremely preterm infants and predict bronchopulmonary dysplasia. A multicenter longitudinal cohort study. Am J Respir Crit Care Med 203:1398–1409 33352083 10.1164/rccm.202008-3131OC

[16] Lichtenstein DA, Mauriat P (2012) Lung ultrasound in the critically ill neonate. Curr Pediatr Rev 8:217–223 23255876 10.2174/157339612802139389 PMC3522086

[17] Loi B, Barra PF, Vivalda L, Raimondi F, De Luca D (2024) Inspiratory-expiratory variation of pleural line thickness in neonates with and without acute respiratory failure. Respir Res 25:12 38178128 10.1186/s12931-023-02651-8 PMC10765855

[18] Parri N, Allinovi M, Giacalone M, Corsini I (2023) To B or not to B. The rationale for quantifying B-lines in pediatric lung diseases. Pediatr Pulmonol 58:9–15 36253340 10.1002/ppul.26185

[19] Lichtenstein DA, Mezière GA (2008) Relevance of lung ultrasound in the diagnosis of acute respiratory Failure*: the BLUE protocol. Chest 134:117–125 18403664 10.1378/chest.07-2800 PMC3734893

[20] Bhalla D, Naranje P, Jana M, Bhalla AS (2022) Pediatric lung ultrasonography: current perspectives. Pediatr Radiol 52:2038–2050 35716179 10.1007/s00247-022-05412-9 PMC9205765

[21] Luca DD, Foti A, Alonso-Ojembarrena A, Condò V, Capasso L, Raschetti R et al (2024) Lung consolidation depth and gas exchange in different types of neonatal respiratory failure: the UNION multicenter study. Chest 165:1431–1434 38367957 10.1016/j.chest.2024.02.012

[22] Lichtenstein D, Mezière G, Seitz J (2009) The dynamic air bronchogram: A lung ultrasound sign of alveolar consolidation ruling out atelectasis. Chest 135:1421–1425 19225063 10.1378/chest.08-2281

[23] Lichtenstein D, Mezière G, Biderman P, Gepner A (2000) The lung point: an ultrasound sign specific to pneumothorax. Intensive Care Med 26:1434–1440 11126253 10.1007/s001340000627

[24] Volpicelli G, Boero E, Sverzellati N, Cardinale L, Busso M, Boccuzzi F et al (2014) Semi-quantification of pneumothorax volume by lung ultrasound. Intensive Care Med 40:1460–1467 25056671 10.1007/s00134-014-3402-9

[25] Copetti R, Cattarossi L (2007) The double lung point: an ultrasound sign diagnostic of transient tachypnea of the newborn. Neonatology 91:203–209 17377407 10.1159/000097454

[26] De Luca D, Bonadies L, Alonso-Ojembarrena A, Martino D, Gutierrez-Rosa I, Loi B et al (2024) Quantitative lung ultrasonography to guide surfactant therapy in neonates born late preterm and later. JAMA Netw Open 7:e2413446 38805223 10.1001/jamanetworkopen.2024.13446 PMC11134216

[27] Brat R, Yousef N, Klifa R, Reynaud S, De Shankar Aguilera S (2015) Lung ultrasonography score to evaluate oxygenation and surfactant need in neonates treated with continuous positive airway pressure. JAMA Pediatr 169:e151797 26237465 10.1001/jamapediatrics.2015.1797

[28] El Amrousy D, Elgendy M, Eltomey M, Elmashad AE (2020) Value of lung ultrasonography to predict weaning success in ventilated neonates. Pediatr Pulmonol 55:2452–2456 32609928 10.1002/ppul.24934

[29] Pang H, Zhang B, Shi J, Zang J, Qiu L (2019) Diagnostic value of lung ultrasound in evaluating the severity of neonatal respiratory distress syndrome. Eur J Radiol 116:186–191 31153563 10.1016/j.ejrad.2019.05.004

[30] Liu J, Copetti R, Sorantin E, Lovrenski J, Rodriguez-Fanjul J, Kurepa D et al (2019) Protocol and guidelines for Point-of-Care lung ultrasound in diagnosing neonatal pulmonary diseases based on international expert consensus. J Vis Exp (145) 10.3791/58990 30907892

[31] Singh Y, Tissot C, Fraga MV, Yousef N, Cortes RG, Lopez J et al (2020) International evidence-based guidelines on point of care ultrasound (POCUS) for critically ill neonates and children issued by the POCUS working group of the European society of paediatric and neonatal intensive care (ESPNIC). Crit Care. 24(1):65. 10.1186/s13054-020-2787-9 PMC7041196 32093763

[32] Mojoli F, Bouhemad B, Mongodi S, Lichtenstein D (2019) Lung ultrasound for critically ill patients. Am J Respir Crit Care Med 199:701–714 30372119 10.1164/rccm.201802-0236CI

[33] Kurepa D, Boyar V, Zaghloul N, Beachy J, Zaytseva A, Teng D et al (2021) Structured neonatal Point-of-Care ultrasound training program. Am J Perinatol 38:e284–e291 32344442 10.1055/s-0040-1709667

[34] Conlon TW, Nishisaki A, Singh Y, Bhombal S, De Luca D, Kessler DO et al (2019) Moving beyond the stethoscope: diagnostic Point-of-Care ultrasound in pediatric practice. Pediatrics 144:e20191402 31481415 10.1542/peds.2019-1402

