# Supplementary material for: Pleura-ABCDE - a structured expert-based protocol for neonatal lung ultrasound documentation and interpretation
Source: Ultrasound J. 2025 Oct 6;17:48. doi: 10.1186/s13089-025-00442-4 (PMC12500510; doi:10.1186/s13089-025-00442-4)
Supplement: Supplementary file 2 — Supplementary Material 2 [file 13089_2025_442_MOESM2_ESM.pdf]

Pleura-ABCDE protocol

Neonatal Lung Ultrasound Examination

**Name**			**Gestational age**	
**Date of birth**			**Corrected gestational age**	

**Breathing support**	no	HFNC	CPAP	nHFO	CMV	iHFO
**Mean airway pressure**						
**FIO2**						
**Indication**						

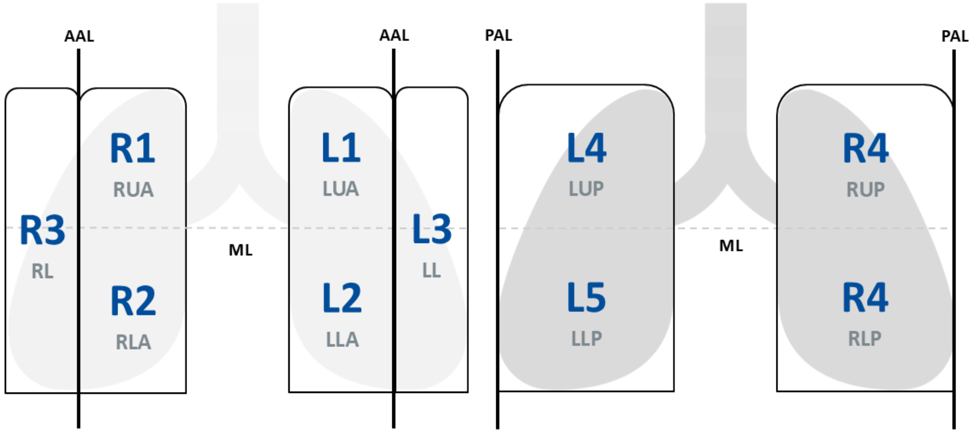

		Right Lung					Left Lung				
		R1	R2	R3	R4	R5	L1	L2	L3	L4	L5
**Pleura**											
	Sliding										
	M-Mode Seashore										
	Stratosphere										
	Morphology Thin										
	Irregular										
**A**	**A-Lines**										
**B**	**B-Lines**										
	Spared										
	Confluent										
**C**	**Consolidation**										
	Size										
	Air Bronchogram										
**D**	**Dynamics**										
	Lung Point										
	Double Lung Point										
**E**	**Effusion**										
	Size										
	**Score**										

**Interpretation**		**Score Σ**
